# Enhanced Antioxidant and Digestive Enzyme Inhibitory Activities of Pacific White Shrimp Shell Protein Hydrolysates via Conjugation with Polyphenol: Characterization and Application in Surimi Gel

**DOI:** 10.3390/foods13244022

**Published:** 2024-12-12

**Authors:** Akanksha R. Gautam, Soottawat Benjakul, Deepak Kadam, Brijesh Tiwari, Avtar Singh

**Affiliations:** 1 International Center of Excellence in Seafood Science and Innovation (ICE-SSI), Faculty of Agro-Industry, Prince of Songkla University, Hat Yai 90110, Songkhla, Thailand; akankshargautam@gmail.com (A.R.G.); soottawat.b@psu.ac.th (S.B.); 2 Department of Food and Nutrition, Kyung Hee University, Seoul 02447, Republic of Korea; 3 Department of Food and Human Nutritional Sciences, University of Manitoba, Winnipeg, MB R3T 2N2, Canada; deepak.kadam@umanitoba.ca; 4 Teagasc Food Research Centre, Ashtown, D15 KN3K Dublin, Ireland; brijesh.tiwari@teagasc.ie

**Keywords:** conjugation efficiency, polyphenols, antioxidant, antidiabetic and *in vitro* digestion

## Abstract

Pacific white shrimp shell protein hydrolysates (SSPHs) produced using alcalase (UAH) and papain (UPH), and polyphenols (PPNs) conjugates were prepared using variable concentrations (0.5–3% *w*/*v*) of different polyphenols (EGCG, catechin, and gallic acid). When 2% (*v*/*v*) of a redox pair was used for conjugation, 0.5% (*w*/*v*) of PPNs resulted in the highest conjugation efficiency (CE), regardless of the polyphenol types. However, CE decreased further with increasing levels of PPNs (*p* < 0.05). SSPHs at 2% retained the highest CE when combined with the selected PPN and redox pair concentrations (*p* < 0.05). FTIR and ^1^H-NMR analysis confirmed the successful conjugation of PPNs with the SSPHs. Among all the conjugates, EGCG conjugated with UAH (A–E) or UPH (P–E) exhibited the highest DPPH/ABTS radical scavenging, and metal chelating activities, respectively. The highest FRAP activity was noticed for A–E conjugate followed by UAH-catechin (A–C) and UPH-catechin (P–C) conjugates. The A–C sample (6 mg/mL) demonstrated the strongest inhibition efficiency against α-amylase, α-glucosidase, and pancreatic lipase (89.29, 81.23, and 80.69%, respectively) than other conjugates (*p* < 0.05). When A–C conjugate was added into surimi gels prepared from Indian mackerel (IM) and threadfin bream (TH) mince at various levels (2–6%; *w*/*w*), gel strength, and water holding capacity was increased in a dose-dependent manner, regardless of surimi type. However, whiteness decreased with increasing A–C levels. After the *in vitro* digestion of surimi gels, antioxidant and enzyme inhibitory activities were also increased as compared to the digest prepared from control surimi gels (added without A–C conjugate). Thus, waste from the shrimp industry in conjugation with plant polyphenols could be utilized to produce antioxidant and antidiabetic or anti-obesity agents, which could be explored as a promising additive in functional foods and nutraceuticals.

## 1. Introduction

Proteins, along with their derivatives, and polyphenols (PPNs) are significant constituents of food, each possessing diverse functional and nutritional qualities. Current scientific inquiry in this area has concentrated on creating bioactive peptides derived from food, particularly emphasizing their health-promoting properties in humans [1]. Protein hydrolysates derived from seafood processing waste are increasingly being recognized as potential ingredients for health-enhancing functional foods, due to their rich content of bioactive peptides and essential amino acids [2]. In addition to showing high nutritional value, hydrolysates possess excellent antioxidant, antidiabetic, and anticancer properties, etc. [3]. Thus, the demand for protein hydrolysates, especially from underutilized food industry waste, remains consistently high. In the shrimp industry, proteins from shrimp shells are typically discarded as waste during the production of chitin and chitosan. These proteins, however, can be recovered and transformed into valuable products, such as protein hydrolysates or bioactive peptides. Nevertheless, protein hydrolysates from shrimp shells have been explored rarely. Recently, in our previous study on peptide fractions from the shrimp shell protein hydrolysates (SSPHs) and their peptides showed excellent antioxidant and antihypertensive activities [4]. Conversely, the production of SSPHs powder resulted in the use of large amounts of maltodextrin (5%) due to their highly hydrophilic nature. Thus, the modification of SSPHs is still required, in which conjugation of SSPHs with plant polyphenols (PPNs) could be an alternative option to tackle the problem. In addition, the bioactivities of the SSPHs could be enhanced further with the conjugation of PPNs [5].

PPNs are a varied group of natural compounds that are widely present in plants. These bioactive molecules are renowned for their antioxidant properties, contributing to numerous health-promoting benefits, such as anti-inflammatory, cardioprotective, diabetes mellitus, and anticancer effects [6]. Among the PPNs, flavonoids, namely epigallocatechin gallate (EGCG) and catechin, as well as phenolic acids like gallic acid, have gained significant attention due to their potent bioactivity and health-promoting potential. Given the diverse functionalities of PPNs, their conjugation with proteins has become a significant area of research focus, particularly when compared to other protein-polymer conjugates. Despite their higher activities, PPNs are prone to oxidation and have low solubility in both hydrophilic and lipophilic systems, limiting their applications [7]. Thus, combining them with various biomolecules such as chitooligosaccharide, starch, protein hydrolysates, etc. could enhance their utility as well as synergistically, they can enhance the various bioactivities of those biomolecules [8]. The fundamental mechanism underlying protein–PPNs conjugation is thought to be oxidation-driven, wherein molecular oxygen oxidizes the hydroxyl group on the PPN to form a quinone [9]. The free radical grafting method is a constructive direction for enhancing the functional attributes and antioxidant capabilities of proteins and involves covalently bonding them with PPN. This method amplifies the presence of hydroxyl groups and hydrophobic regions, thereby potentially altering or enhancing the protein’s bioactivities and functional properties [10].

Surimi, a processed fish product widely used in food applications, relies heavily on protein gelation and texture for consumer acceptance [11,12]. Improving the functional properties of surimi by incorporating bioactive conjugates such as protein-PPN conjugates could enhance its commercial value. Protein hydrolysates or PPNs are known to enhance the interactions among surimi proteins via various bondings including hydrophobic, covalent, hydrogen, etc., resulting in enhanced textural properties. The high sugar content in surimi, which is used as a cryoprotectant, could create health-related problems such as diabetes [13]. Therefore, applying antidiabetic peptides or protein hydrolysate conjugated with the PPN could act as antidiabetic agents by inhibiting the digestive enzymes, especially α-amylase and α-glucosidase.

Thus, this study seeks to explore the potential benefits of modifying protein through conjugation with EGCG, catechin, and gallic acid. By examining the structural and functional characteristics of these conjugates, we aim to elucidate their potential applications in surimi gel from different sources such as dark-flesh surimi (Indian mackerel) and white-flesh surimi (threadfin bream).

## 2. Materials and Methods

### 2.1. Raw Material and Chemicals

Pacific white shrimp (*Litopenaeus vannamei*) shells were sourced from Sea Wealth Frozen Food in Songkhla, Thailand. Food grade alcalase (EC 3.4.21.62: 124 units/mL) and papain (E.C. 3.4.22.2; 379 units/mL) from *Bacillus licheniformis* and *Carica papaya* latex, respectively, were obtained from Siam Victory Chemicals Co., Ltd. (Bangkok, Thailand). EGCG, gallic acid, and catechin were procured from Xi’an Julong Bio-Tech Co., Ltd. (Xi’an, China). The chemicals and enzymes used were also acquired from Sigma-Aldrich (St. Louis, MO, USA).

### 2.2. Preparation of Protein Hydrolysates from Shrimp Shells (SSPHs)

SSPHs were derived from the shrimp shell using our previously optimized conditions by Gautam et al. [14]. Demineralization (1 M HCl; 1:20 *w*/*v*) for 1 h and shrimp shell protein (SSP) were recovered using 1 M NaOH (1:30 *w*/*v*) at 60 °C for 1 h. Then, 2 g of freeze-dried SSP powder was dissolved in 100 mL of distilled water and pretreated with optimized ultrasonication conditions (60% amplitude for 15 min). Thereafter, the pretreated solution was subsequently hydrolyzed using 2% (*v*/*v*) alcalase and 3% (*v*/*v*) papain for 90 min at 60 °C and 50 °C, respectively. The resultant shrimp shell hydrolysates (SSPHs) prepared using alcalase (UAH) and papain (UPH) were recovered by centrifugation at 20,000× *g* for 15 min at 4 °C and freeze-dried for further applications.

### 2.3. Conjugation of Shrimp Shell Protein Hydrolysate with Different Polyphenols

SSPH–PPNs were derived using free radical grafting following the method of Chotphruethipong et al. [15]. From the preliminary study, SSPHs (UAH and UPH) and redox radical concentrations (2% for both) were fixed. Firstly, 2 g of UAH or UPH were dissolved in 100 mL distilled water. Simultaneously, redox pair solution was prepared by incubating 0.15 g of ascorbic acid in 5 M H_2_O_2_ for 30 min under dark conditions to generate radicals at 27 °C, which was mixed with UAH or UPH at 2% (*v*/*v*) for 2 h at 27 °C under in dark conditions. Thereupon, PPNs (EGCG, catechin, or gallic acid) at 0.5, 1, 2, and 3% (*w*/*v*) were added to the mixtures and stirred for 24 h at 27 °C. The resultant mixture was further subjected to dialysis (cut-off 500 Da) at 4 °C for 24 h against the distilled water to remove unbound PPNs. The conjugation efficiency (CE) was determined by analyzing the total phenolic content (TPC) using Folin–Ciocalteu reagent (Equation (1)).

(1) $$\mathrm{CE}\;\left(\%\right)=\frac{\mathrm{Concentration}\;\mathrm{of}\;\mathrm{PPN}\;\mathrm{in}\;\mathrm{UAH}/\mathrm{UPH}\;\mathrm{conjugate}}{\mathrm{Initial}\;\mathrm{concentration}\;\mathrm{of}\;\mathrm{PPN}\;\mathrm{used}\;\mathrm{for}\;\mathrm{conjugation}}\times 100$$

PPN concentrations showing the highest CE were selected for further study. UAH or UPH conjugated with a selected level of EGCG or catechin or gallic acid showing the highest CE was freeze-dried and named as A–E, A–C, and A–G, or P–E, P–C, and P–G, respectively.

### 2.4. Characterization of Selected Shrimp Shell Protein Hydrolysate and Polyphenol Conjugates

#### 2.4.1. Fourier-Transform Infrared (FTIR) Spectra

Freeze-dried samples were subjected to FTIR detection. An Equinox 55 FTIR spectrometer (Bruker, Ettlingen, Germany), equipped with an attenuated total reflection (ATR) diamond crystal cell, was utilized for this purpose. Spectra were collected over the wavenumber range of 400–4000 cm^−1^, with 32 scans at a resolution of 4 cm^−1^ at 25 °C, and analyzed using OPUS 3.0 software (Bruker, Ettlingen, Germany).

#### 2.4.2. ^1^H-NMR

^1^H-NMR spectra of samples were recorded in D_2_O and chemical shifts were expressed as parts per million (ppm) following the method of Huang et al. [8]. The 200 μL samples dissolved D_2_O were transferred into 3 mm NMR tubes and analyzed using a 500 MHz Fourier-transform NMR spectrometer (Bruker AVANCE NEO 500 MHz, Ettingen, Germany) operated at 500.15 MHz. NMR spectra were processed for baseline correction, phase adjustment, and calibrated to the residual D_2_O solvent peak.

#### 2.4.3. Antioxidant Activities

Antioxidant activities, including DPPH-radical scavenging activities (DPPH-RSA) and ABTS-radical scavenging activities (ABTS-RSA), ferric reducing antioxidant power (FRAP), and metal chelating activity (MCA) of all the conjugate samples were determined using the methods described below [16].

The DPPH-RSA was measured by combining 1.5 mL of sample solution with 1.5 mL of 0.1 mM DPPH solution in 95% ethanol. This mixture was then incubated for 30 min at room temperature in darkness. After incubation, the absorbance was read at 517 nm. A control was prepared under identical conditions, substituting the sample with distilled water. The activity was quantified using a Trolox standard curve (0–60 µM) and expressed as μmol Trolox equivalents (TE)/gram of solids.

For ABTS-RSA, a stock solution of 14.8 mM ABTS and 5.2 mM potassium persulfate was used. Equal volumes of the stock solutions were mixed and allowed to react for 12 h in the dark at room temperature, creating the working solution. This solution was then diluted with methanol to achieve an absorbance of 1.1 ± 0.02 at 734 nm. The activity was determined using a Trolox standard curve, ranging from 0 to 600 μM, and expressed as μmol TE/gram of solids.

The FRAP assay involved mixing a freshly prepared FRAP reagent, composed of 10 mM 2,4,6-Tris(2-pyridyl)-s-triazine (TPTZ) in 40 mM HCl, 20 mM FeCl_3_·6H_2_O, and 300 mM acetate buffer at pH 3.6 (in a ratio of 1:1:10, *v*/*v*/*v*). This reagent (2.85 mL) was pre-incubated at 37 °C for 30 min. Then, 150 μL of the sample was added, and the mixture was left in the dark at room temperature for 30 min. The absorbance was measured at 593 nm. The activity was calculated based on a Trolox standard curve (0–600 μM) and expressed as μmol TE/gram of solids.

MCA was assessed by mixing 200 μL of the sample with 800 μL of distilled water, followed by the addition of 0.1 mL of 2 mM FeCl_2_ and 0.2 mL of 5 mM ferrozine. The reaction mixture was allowed to stand at room temperature for 20 min, and absorbance was measured at 562 nm. An EDTA standard curve was used, ranging from 0 to 30 μM, to express chelating activity as μmol EDTA equivalents (EE)/gram of solids. A blank was prepared in the same manner, replacing the sample with distilled water.

#### 2.4.4. Digestive Enzyme Inhibitory Activity

The enzyme inhibitory activity of the selected conjugate against α-amylase, α-glucosidase, and lipase was determined using starch, para-nitrophenyl glycoside, and 4-methylumbelliferyl oleate as a substrate as per the methods described by Silva et al. [17].

##### α-Amylase Inhibitory Activity

The evaluation of α-amylase inhibition was conducted using soluble starch as the substrate. Conjugate samples were prepared at concentrations of 2, 4, and 6% (*w*/*v*) in 1 mL of 20 mM sodium phosphate buffer (pH 6.9) containing 6 mM NaCl. To each test tube, 100 μL of the sample solution and 100 μL of α-amylase solution (100 μg/mL) were combined and incubated at 25 °C for 10 min. Subsequently, 100 μL of 1% (*w*/*v*) starch solution, also prepared in the same buffer, was added, and incubated again at 25 °C for 10 min. To terminate the reaction, 200 μL of dinitrosalicylic acid (DNSA) color reagent (consisting of 96 mM DNSA, 2 M sodium potassium tartrate tetrahydrate, and 2 M NaOH) was added, and the mixtures were heated in a boiling water bath (100 °C) for 5 min. After cooling to room temperature, 3 mL of double-distilled water was added to each mixture. Absorbance was then recorded at 540 nm using a FLUOstar^®^ Omega microplate reader (BMG Labtech, Ortenberg, Germany) maintained at 25 °C. A blank sample, in which the enzyme was replaced by the buffer, was used for baseline correction. Acarbose, a standard α-amylase inhibitor, served as a positive control and followed the same procedure. The percentage of inhibition was calculated using Equation (2).

Inhibition (%) = [1 − ((As − Asb)/Ac)] × 100(2)

As, Asb, and Ac, absorbance of sample, absorbance of blank and absorbance of control, respectively.

##### α-Glucosidase Inhibitory Activity

Firstly, the α-glucosidase enzyme source was prepared by homogenizing 500 mg of rat intestinal acetone powder in 9 mL of 0.9% NaCl solution and centrifuged at 12,000 ×g for 30 min. The resulting supernatant served as the enzyme source. Conjugate samples were prepared at concentrations of 2, 4, and 6% (*w*/*v*) in 0.1 M sodium phosphate buffer (SPB) at pH 6.9. A 50 μL aliquot of each sample was mixed with 50 μL of α-glucosidase enzyme solution (10 mg/mL) in a 96-well microtiter plate and incubated at 37 °C for 10 min. Following this, 100 μL of 5 mM PNP-glycoside solution (prepared in 0.1 M SPB, pH 6.9) was added to the wells containing the sample-enzyme mixture and incubated at 37 °C for an additional 30 min. The absorbance of the reaction was recorded at 405 nm using a microplate reader. A blank sample was prepared by omitting the enzyme from the reaction mixture. The α-glucosidase activity was determined based on the release of p-nitrophenol from PNP-glycoside. Acarbose, prepared at the same concentrations as the sample, was used as a positive control. Control reactions, using double-distilled water instead of the sample, were also conducted. The percentage inhibition of α-glucosidase activity was calculated using Equation (3).

Inhibition (%) = [1 − ((As − Asb)/Ac)] × 100(3)

Ac, As, and Asb represent the absorbance of the control, sample, and sample blank, respectively.

##### Lipase Inhibitory Activity

To determine pancreatic lipase inhibitory activity, conjugates at various levels (2, 4, and 6%; *w*/*v*) were prepared in tris buffer (13 mM Tris-HCl, 150 mM NaCl, and 1.3 mM CaCl_2_; pH 8.0). Each 25 μL sample solution was mixed with 225 μL of 0.5 mM 4-MU oleate solution in a 96-well microplate and incubated at 37 °C for 15 min. Following this, 25 μL of pancreatic lipase solution (50 U/mL) was added to each well, and the mixture was incubated for 1 h at 37 °C. The amount of 4-MU released, indicative of lipase activity, was measured at 400 nm using a microplate reader. Orlistat, a known pancreatic lipase inhibitor, was used as a positive control at the same concentrations. Control reactions were prepared by replacing the sample with double-distilled water. The percentage of pancreatic lipase inhibition was calculated using Equation (4):

Inhibition (%) = [1 − ((As − Asb)/Ac)] × 100(4)

Ac, As, and Asb indicate absorbance of the control, sample, and sample blank, respectively.

The UAH conjugated with catechin (A–C) showed the highest inhibitory activity against the digestive enzymes, which was selected for further application in gel prepared from TH and IM surimi.

### 2.5. Effect of A–C Conjugate on the Gel Properties of Threadfin Bream and Indian Mackerel Surimi Gel

#### 2.5.1. Preparation of Surimi

Threadfin bream (TH) (*Nemipterus* sp.) and Indian mackerel (IM) (*R. kanagurta*), each weighing between 100–120 g/fish, were sourced from a local fish market in Hat Yai, Songkhla, Thailand. Both species were transported on ice, maintaining a fish-to-ice ratio of 1:2 (*w*/*w*), and promptly delivered to the seafood laboratory. Upon arrival, the fish were rinsed with cold water, beheaded, and eviscerated. The flesh was manually separated from the bones and minced using a grinder. The minced flesh was then washed in cold water (5–8 °C) at a ratio of 1:3 (*w*/*v*) mince-to-water and filtered through cheesecloth. This washing process was repeated three times to ensure purity. After washing, the mince was centrifuged at 700× *g* for 15 min in a basket centrifuge (Model CE 21 K, Grandiumpiant, Belluno, Italy). The washed mince was then combined with cryoprotectant (4% sorbitol and 4% sucrose), packed in polyethylene bags, and frozen at −20 °C. The surimi prepared from both fish was stored and utilized within one month.

#### 2.5.2. Preparation of Surimi Gels

Frozen surimi was thawed under running tap water until the core temperature reached 0–2 °C and was cut into small cubes (1 × 1 × 1 cm^3^). Thereafter, 150 g of surimi was blended for 1 min followed by the addition of 2.5% (*w*/*w*) of salt and further blending of 1 min. The surimi paste was added with the A–C conjugate at various concentrations of 2, 4, and 6% (*w*/*w*). Finally, the moisture content of the surimi paste was adjusted to 80% (*w*/*w*) by adding cold water and the paste was blended further. Overall, blending was performed for 4 min and the temperature of the paste was maintained below 10 °C. The prepared paste was then stuffed into polyvinylidene chloride casings, each with a diameter of 2.0 cm. Both ends of the casings were tightly sealed, and the surimi paste was set at 40 °C for 30 min, followed by cooking at 90 °C for 20 min. Thereafter, the gels were immediately cooled in ice water for 60 min and stored at 4 °C for 15–18 h before analysis [18].

### 2.6. Analyses

#### 2.6.1. Gel Properties

Gel properties including breaking force (BF), deformation (DF), whiteness, and expressible moisture content (EMC) were determined by following the methods of Ma et al. [12].

In brief, the BF and DF of surimi gels were measured using a texture analyzer (Model TA-XT2, Stable MicroSystems, Godalming, Surrey, UK) fitted with a 5 mm spherical plunger. The BF, which refers to the force required to puncture the gel, and the DF, which is the distance the plunger was penetrated the gel, were recorded.

The whiteness of gel samples was determined using a colorimeter (HunterLab, Colorflex, Hunter Associates Laboratory, Reston, VA, USA). CIE L*, a*, and b* values were measured, and whiteness was then calculated using Equation (5):

Whiteness = 100 − [(100 − L*)^2^ + a*^2^ + b*^2^]^1/2^ (5)

where L* is the lightness; a* is the redness/greenness; and b* is the yellowness/blueness.

For EMC, cylindrical gel samples (5 mm in thickness and weighing X g) were positioned between three layers of Whatman filter paper No. 1 at the bottom and two layers on the top. A standard weight of 5 kg was applied to the top of the sample for 2 min, after which the weight of the sample (Y) was measured. The EMC was then calculated using the following formula:

EMC (%) = [(X − Y)/X] × 100(6)

#### 2.6.2. Texture Profile Analysis (TPA)

The gel samples were assessed for hardness, springiness, cohesiveness, gumminess, and chewiness using a texture analyzer (Model TA-XT2, Stable MicroSystems, Surrey, UK) equipped with a cylindrical probe (35 mm diameter) [18].

#### 2.6.3. Protein Patterns

The protein patterns of surimi gels were analyzed using sodium dodecyl sulfate-polyacrylamide gel electrophoresis (SDS-PAGE) following the method by Azadian et al. [19]. The patterns were obtained on 10% (*w*/*v*) polyacrylamide running gel and a 4% (*w*/*v*) stacking gel for electrophoresis.

#### 2.6.4. Scanning Electron Microscopic (SEM) Images

The concentration of A–C conjugate that provided the best gel strength and texture profile in IM and TH surimi gels were visualized using a field emission-scanning electron microscope (FE-SEM) (Apreo, FEI, Amsterdam, The Netherlands). The fixation and drying of the samples were performed following the method of Mi et al. [20]. The sample with a thickness of 2–3 mm was dehydrated using ethanol with serial concentrations (25–100%) before fixing with 2.5% (*v*/*v*) glutaraldehyde (prepared in 0.2 M phosphate buffer; pH 7.2) for 3 h at room temperature. Then, the samples were critical point dried, followed by sputter coating with gold, and visualized using SEM.

#### 2.6.5. *In Vitro* Gastrointestinal Digestion of Surimi Gel

The surimi gel samples were subjected to gastric and duodenal digestion as following the method explained by Fang et al. [21]. The surimi gel samples were first homogenized with distilled water at a ratio of 1:10 (*w*/*v*) and then mixed with freshly prepared pepsin solution (4% in 0.1 M HCl). The gastric phase of digestion was simulated by adjusting the pH to 2.0, and the mixture was incubated at 37 °C for 60 min in a water bath shaker. To mimic the duodenal phase, the pH was raised to 6.8 using 0.9 M sodium bicarbonate, followed by the addition of 0.3 mL of freshly prepared 4% bile salts and 0.08% pancreatin. Digestion continued at 37 °C for 120 min in the water bath shaker. The reaction was stopped by boiling the samples for 2 min, after which they were cooled in ice water. All samples were then centrifuged at 8500× *g* for 10 min, and the supernatant was collected for analysis.

##### Digestive Enzyme Inhibitory

The antidiabetic potential of *in vitro* digest was evaluated through their ability to inhibit key digestive enzymes, including α-amylase, and α-glucosidase. Furthermore, lipase inhibitory activity was also measured as previously mentioned.

##### Antioxidant Activity

Antioxidant activities, including DPPH-RSA and ABTS-RSA of digest samples were examined following the previously explained.

### 2.7. Statical Analysis

Each assay was conducted in triplicate, and the data were reported as mean values ± standard deviation. Statistical analysis was carried out using analysis of variance (ANOVA), followed by Duncan’s multiple range test to compare data sets, with significance established at *p* < 0.05. All statistical analyses were performed using SPSS software (SPSS 22 for Windows, SPSS Inc., Chicago, IL, USA).

## 3. Results and Discussion

### 3.1. Effect of Varying Concentrations of AsA/H_2_O_2_ Redox Pair Initiators on Conjugation Efficiency (CE)

The conjugation efficiency (CE) of SSPHs (UAH or UPH) conjugated with 2% (*w*/*v*) of EGCG, catechin, and gallic acid at varying concentrations of redox pair is summarized in Table 1. The results showed significant increases in CE with increasing concentration of redox pair, irrespective of the type of PPN used (*p* < 0.05). Notably, the highest CE was noticed at 2% of redox pair concentration (82–95%). In general, hydroxyl radicals (HO^˙^) generated during the free radical grafting process attack hydrogen atoms at hydroxyl, amino, or sulfhydryl sites on the protein, leading to the formation of intermediate products (macro-radicals of UAH or UPH) that subsequently form complexes with PPNs [22], due to the ability of PPNs to stabilize radicals. Hence, with increasing concentrations of redox pair, a higher amount of hydroxyl radicals (HO^˙^) were produced. These radicals attack hydrogen atoms at hydroxyl, amino, or sulfhydryl sites on the protein, leading to the formation of intermediate products (macro-radicals) that subsequently form complexes with PPNs [22]. As the redox pair concentration increases, so does the number of macro-radicals of UAH or UPH, thereby increasing the probability of these radicals interacting with PPNs to form conjugates [7]. Among all the PPNs studied, conjugates prepared using gallic acid with UAP or UAH exhibited the highest CE (95.41 or 94.40%, respectively). This was more likely due to the smaller structure of gallic acid as compared to EGCG and catechin, leading to lower hindrance in the covalent binding with the macro radicals of UAH/UPH [23]. These differences in CE are often associated with variations in individuals’ molecular weight, the number of free radical binding sites, and phenolic content. A similar finding was noticed by Liu et al. [7], where whey protein isolate was conjugated with EGCG, quercetin, apigenin, and naringenin. EGCG resulted in higher CE (87.02 ± 4.36%), in comparison to quercetin, apigenin, and naringenin (70.24 ± 5.18, 65.51 ± 3.78 and 57.80 ± 0.76%, respectively). Similarly, Mittal et al. [24] noticed differences in CE when different polyphenols were conjugated with chitooligosaccharide, further supporting the notion that structural characteristics of PPNs play a critical role in their conjugation behavior.

Based on these findings, the 2% redox pair concentration was optimal for further study, given its significant impact on improving CE across all polyphenols tested.

### 3.2. Effect of Different Concentrations of SSPHs and PPNs on the Conjugation Efficiency

The CE of different SSPHs and PPNs at varying concentrations is presented in Table 1. It has been observed that as the concentration of PPNs increased, the CE progressively decreased, regardless of the type of PPN used. This pattern suggests that increasing the concentration of PPNs beyond 0.5% did not lead to a further enhancement of CE. This can be attributed to several factors related to protein structure, molecular conformation, and the availability of binding sites [25]. Higher PPN concentrations can result in the saturation of available binding sites, limiting effective conjugation [26]. As a result, excess PPN molecules may compete for limited binding sites on the protein, decreasing the overall CE [27]. At lower concentrations of PPNs (0.5%), the binding sites on the protein are optimally available, allowing for the maximum conjugation with the different PPNs. Furthermore, at higher concentrations, protein hydrolysates or PPN molecules may begin to compete for the limited number of available binding sites [28]. This competition may limit effective conjugation as more PPN molecules vie for the same protein binding sites, reducing the overall CE [29]. These findings align with the study of Chotphruethipong et.al [15], where hydrolyzed collagen conjugated with varying concentrations of EGCG (1–5%) showed an increase in CE up to 97.26% as the EGCG concentration reached 3%. However, further increases in EGCG concentration led to a decrease in CE to 96.02%. Among all the PPNs studied, conjugates prepared using gallic acid with UAP or UAH exhibited the highest CE (*p* < 0.05). This was more likely due to the smaller structure of gallic acid as compared to EGCG and catechin, leading to lower hindrance in the covalent binding with the macro radicals of UAH/UPH [30]. 

When 0.5% PPN concentration was used to conjugate with SSPHs at different levels, the highest CE was noticed for 2% SSPHs (*p* < 0.05) (Table 1). Where with further increasing levels to 3%, no change in CE was noticed (*p* < 0.05). The increasing levels of SSPHs might provide a higher number of macro-radicals of SSPHs, which were able to bind with PPNs. However, higher levels of SSPHs might not result in the formation of excessive macro-radicals or generated macro-radicals might be bound with all PPNs. Furthermore, as previously explained, due to the limited number of macro-radicals or hindrances provided by the bound PPNs due to their structure difference could be another possible reason for no change in the CE.

Considering the CE, the optimal concentration for redox pair, PPNs, and SSPHs were 2%, 0.5%, and 2%, were used for further study.

### 3.3. Characterization of Protein Hydrolysate and Polyphenol Conjugates

#### 3.3.1. FTIR Spectra

**Figure 1:**
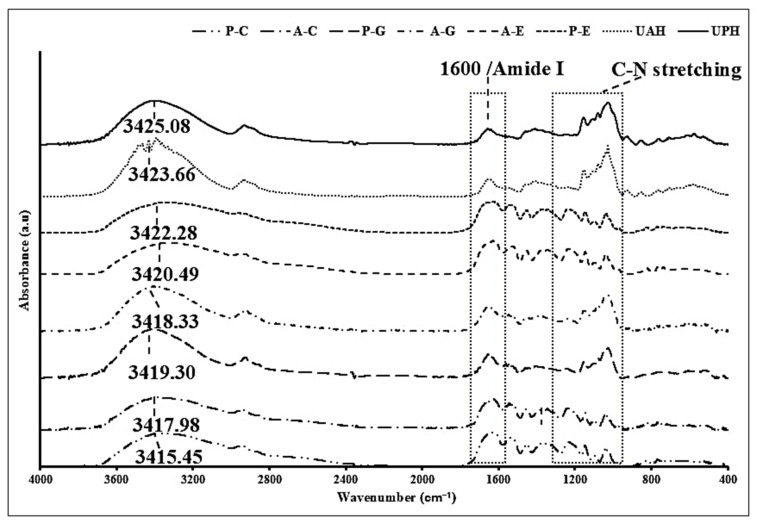
FTIR spectra of shrimp shell protein hydrolysate (SSPH) prepared using alcalase (UAH) or papain (UPH) and their polyphenol conjugates. Where A and P indicate UAH and UPH; E, C, and G indicate EGCG, catechin; and gallic acid, respectively.

The FTIR spectra provides key insights into the structural changes that occurred due to the conjugation of SSPHs with PPNs as shown in Figure 1. In the SSPH spectra, the characteristic bands observed around 3500 cm^−1^ are associated with O-H stretching vibrations, which indicate the presence of hydroxyl groups [31]. Similarly, the peaks in the range of 2800–3000 cm^−1^ correspond to C-H stretching vibrations, typically found in aliphatic compounds [32]. The presence of peaks around 1600 cm^−1^ is indicative of N-H bending vibrations in primary amines, suggesting the presence of protein-derived functional groups such as amines and amides [32]. Moreover, the spectral region between 1000 and 1200 cm^−1^ corresponds to C-N stretching vibrations, further confirming the proteinaceous nature of SSPHs [22].

Upon conjugation with PPNs, noticeable shifts and broader spectra were observed. The broad band in the range of 2400–3800 cm^−1^ suggests the formation of extensive hydrogen bonds between the hydroxyl groups of PPNs and the amine or amide groups of the SSPHs. Compared with UAH and UPH (SSPHs), amide A bands of A–E, A–C, A–G, P–E, P–C, and P–G conjugates had wider peak shapes, and their maximum absorption wave number was shifted to 3300 to 3700 cm^−1^, respectively, indicating that the PPNs covalently binding to SSPHs, which could strengthen the stability and bioactivity of the conjugated product (Figure 1) [30]. In particular, the appearance of peaks related to C=O stretching vibrations, which are common in ester and carbonyl groups, suggests the occurrence of covalent bonding between PPNs and SPPHs [28]. Supporting the notion of results, similar structural change observation was reported by Pan et al. [26] during the fabrication of conjugates between different PPNs and bovine bone proteins.

#### 3.3.2. ^1^H-NMR Spectra

**Figure 2:**
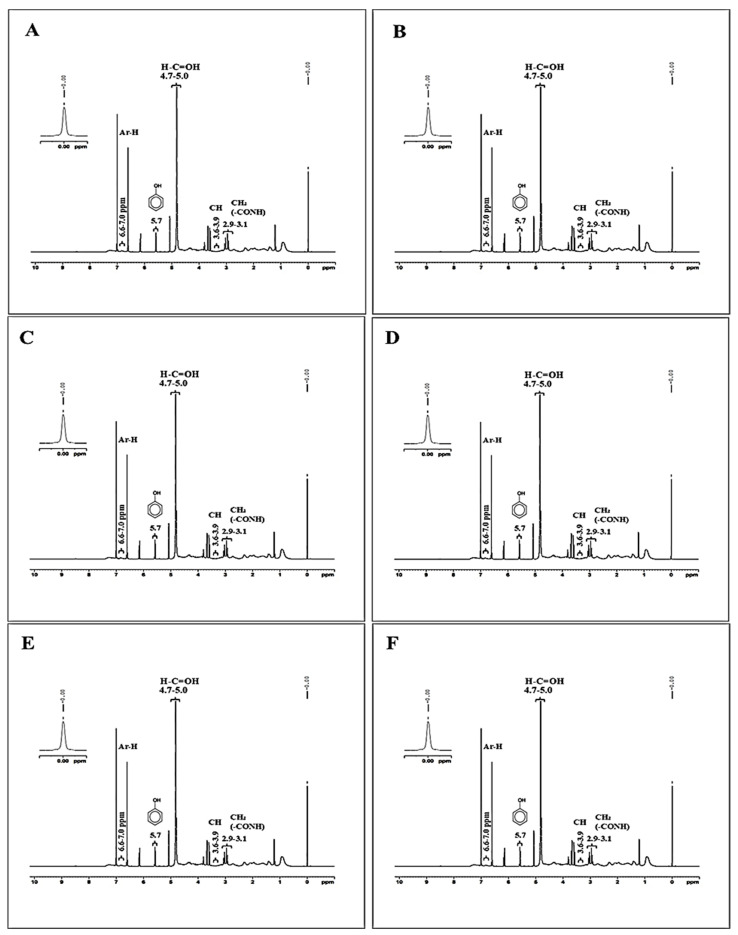
^1^H-NMR spectra of shrimp shell protein hydrolysate (SSPH) prepared using alcalase (UAH) or papain (UPH) and their polyphenol conjugates. A–C (**A**), P–C (**B**), A–G (**C**) P–G (**D**), A–E (**E**) and, and P–E (**F**). Abbreviations: see Figure 1.

^1^H-NMR spectra of selected samples are illustrated in Figure 2A–F. Signals at 2.9–3.1 ppm can be attributed to the aliphatic hydrogens adjacent to electronegative atoms, likely present in the protein side chains or hydrolysate backbone [29]. Moreover, the methylene (CH_2_) groups are near amide or hydroxyl functionalities introduced through the conjugation process. Whereas 3.6–3.9 ppm signal region typically corresponds to methine (CH) hydrogens attached to oxygen or nitrogen atoms. In the context of the conjugate, these signals are likely derived from the sugar rings of gallic acids or EGCG or catechin [29]. Additionally, a signal of 4.7–5.0 ppm is associated with anomeric protons or hydrogens attached to carbons bearing hydroxyl groups. These shifts suggest that the gallic acid, EGCG and catechin components have maintained their sugar ring structures upon conjugation, and the hydroxyl groups on these rings remain in an environment influenced by their aromatic cores. Signal at 5.7 ppm is characteristic of hydrogens in conjugated systems, particularly those involving aromatic rings with hydroxyl groups, whereas peaks at 6.6 ppm and 7.0 are indicative of aromatic hydrogens on benzene rings [31]. The signals in this range confirm the presence of aromatic structures from gallic acid, EGCG, and catechin within the conjugate. The distinct aromatic proton signals suggest that these polyphenolic compounds have been successfully grafted onto the SSPHs, retaining their aromatic nature.

### 3.4. Antioxidant Activities of SSPHs and SSPH–PPNs

The antioxidant activity of the SSPH-PPNs showed a significant enhancement (*p* < 0.05) compared to SSPHs (UAH and UPH) (Table 2). Among UAH and UPH, the former had the highest ABTS-RSA and MCA activities, and the latter one showed the maximum value for the remaining assays tested (*p* < 0.05). The difference in activities was mainly associated with the amino acid present in their polypeptide chain, which is influenced by the enzyme’s specificity for proteins. In general, protein hydrolysates are known as excellent antioxidant agents, which is more likely due to the presence of smaller peptide fragments and free amino acids that often have functional groups (e.g., -NH_2_, -SH) capable of donating hydrogen atoms or electrons [33]. These peptides, especially those rich in amino acids such as histidine, tyrosine, cysteine, and tryptophan, play an important role in scavenging free radicals [34]. The increased antioxidant activities after conjugation, irrespective of the PPN type, underscores the synergetic effects between the protein hydrolysates and PPNs, leading to higher radical scavenging activities [35]. Among all conjugates, EGCG conjugated SSPHs had the highest DPPH-RSA, ABTS-RSA, and MCA activities than the remaining PPNs. Whereas gallic acid and SSPHs conjugates showed lower activity than the remaining conjugates (*p* < 0.05). This could be associated with multiple hydroxyl groups across EGCG, particularly on the B and D rings, which also stabilize free radicals after donation than the other PPNs, especially gallic acid with only one aromatic ring and three hydroxyl groups. The increased DPPH and ABTS activities suggest that the conjugate has a higher capacity to donate hydrogen atoms to neutralize free radicals, which is a principal aspect of the antioxidant defense mechanism. Similarly, Liu et.al [7] reported protein-PPN conjugates showed improved radical scavenging activities. Additionally, MCA observed their capacity to bind metal ions, which are known to catalyze oxidative reactions via acting as prooxidants [10]. The FRAP assay revealed higher activity in A–E, followed by A–C and P–C conjugates. This result indicates the superior ability of the A–E conjugates to reduce ferric ions, highlighting their potential as strong reducing agents, which could be related with the complex structure of EGCG. Whereas PPNs are well-known for their strong antioxidant effects, which help to neutralize radicals and reduce oxidative stress. The effects arise from their ability to donate hydrogen atoms or electrons, effectively scavenging reactive oxygen species and other free radicals [35].

Thus, the synergetic effect between protein hydrolysates and PPNs represents a promising strategy for enhancing the bioactivity of proteins for potential nutraceutical or food applications. Furthermore, conjugates with different antioxidant activities could allow them to be used in the different food systems.

### 3.5. Antidiabetic Activity of Protein Hydrolysate and Polyphenol Conjugates

α-Amylase Inhibitory Activity

**Figure 3:**
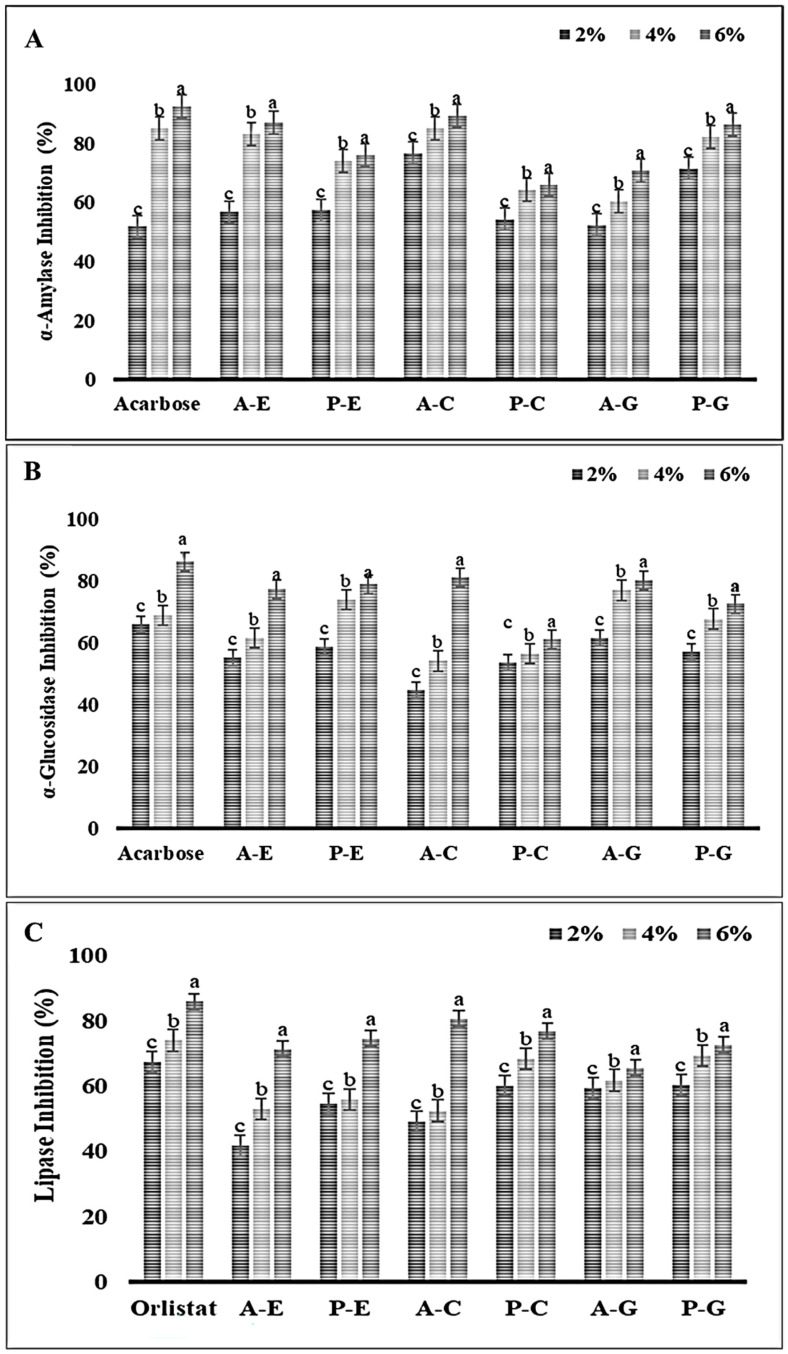
Inhibitory activity of shrimp shell protein hydrolysate (SSPH) and their polyphenol conjugates against various digestive enzymes such as α-amylase (**A**), α-glucosidase (**B**), and pancreatic lipase (**C**). Bars represent standard deviation (n = 3). Lowercase letters on the bars indicate a significant difference between the different concentrations of the same sample (*p* < 0.05). Where 2, 4, and 6 represent the concentration (%; *w*/*v*) of the conjugates used. Abbreviations: see Figure 1.

α-Amylase plays an important role in the digestion of dietary starch, breaking it down into oligosaccharides that are further hydrolyzed into glucose, which is quickly absorbed by the body. Therefore, the inhibition of such enzymes is considered to be a viable approach for managing diabetes as well as linked diseases such as obesity, hypertension, etc. [36]. As shown in Figure 3A, all SSPH–PPN conjugates at each tested concentration showed inhibition against α-amylase. With the increase in concentration of conjugates, the inhibition activity also increased, the conjugate at 6 mg/mL exhibited the highest inhibitory activity (*p* < 0.05). At a concentration of 6 mg/mL, α-amylase inhibitory activities for A–E, A–C, A–G, P–E, P–C, and P–G were 87.03%, 89.27% 70.9%, 75.92%, 66.04%, and 86.50%, respectively. The higher concentration of conjugates more likely inhibits enzyme activity by forming hydrogen bonds or ionic interactions with the amino acid residues at the enzyme’s active site, leading to structural changes in the enzyme. These stronger binding interactions result in greater inhibitory activity, as the structural modifications interfere with the enzyme’s normal function [37]. Notably, among all conjugates, A–C showed the highest inhibition, which is probably due to high binding affinity with the active enzyme’s site [31]. These findings align with the screening study of PPN inhibition against α-amylase and α-glucosidase by Rasouli et al. [38], which identified catechin as a potential inhibitor of α-amylase. Similarly, Uraipong et.al [39] reported that the rice bran protein hydrolysates had α-amylase inhibition properties. Thus, PPNs, especially catechin and peptides derived from hydrolysate, have promising potential as natural inhibitors of α-amylase, offering a novel approach to diabetes management through dietary interventions.

α-Glucosidase Inhibitory Activity

α-glucosidase is an enzyme located in the epithelium of the small intestine, which expedites the breakdown of oligo- and disaccharides into glucose [40]. Inhibiting this enzyme is a well-known approach to reducing serum glucose levels. Thus, the outcomes of the rat intestinal α-glucosidase inhibition by the samples are shown in Figure 3B. Like the α-amylase inhibitory activity study, all conjugates demonstrated a concentration-dependent inhibitory activity. The lowest conjugates’ tested concentration (2 mg/mL) expressed the lowest inhibition activity, whereas as 6 mg/mL concentration of all conjugates had the highest inhibition activity against α-glucosidase (A–E, A–C, A–G, P–E, P–C, and P–G 77.51%, 81.23%, 80.39%, 79.06%, 61.24%, and 72.67, respectively). A similar result was noticed by Yang et.al. [41], when α-glucosidase was inhibited by green tea PPNs in a dose-dependent manner. These studies reinforce the notion that higher concentrations of PPN conjugates can interact more effectively with the enzyme, potentially forming stronger bonds with the active site, and leading to enhanced inhibition. Similarly, peptides derived from various sources of protein hydrolysates have shown promising inhibitory effects on α-glucosidase, an enzyme that catalyzes the final steps in the digestion of carbohydrates, converting disaccharides into glucose for rapid absorption [42]. This alignment with other studies suggests that SSPH–PPNs conjugates could serve as potent natural inhibitors of α-glucosidase, contributing to the development of functional foods or nutraceuticals aimed at managing blood glucose levels and related abnormalities.

Lipase Inhibitory Activity

Pancreatic lipase is critical for the digestion of dietary triacylglycerols in the intestine, a dominant contributor to surplus caloric intake. Enhanced lipid absorption in the pancreas can stimulate the insulin-producing β-cells, potentially leading to the onset of type

I and type II diabetes [43]. The obstruction or interference in digestion and absorption of lipids through the suppression of lipase activity is a pivotal target. Like α-amylase and α-glucosidase inhibition, the prominent trend in inhibition activity was exhibited by the increasing SSPH–PPNs concentration (Figure 3C). The highest lipase inhibitory activities for A–E, A–C, A–G, P–E, P–C, and P–G conjugates observed at 6% (mg/mL) of concentration, were 71.34%, 80.69%, 65.6%, 74.59%, 76.82% and 72.67%, respectively. The lipase inhibitory activity of these conjugates suggests that their interaction with the enzyme may involve binding to the lipase’s active site, likely forming hydrogen bonds or ionic interactions with critical residues, thereby disrupting the enzyme’s ability to hydrolyze triacylglycerols [44]. Peptides derived from various protein hydrolysates have demonstrated diverse bioactivities, as highlighted by findings showing that de-oiled rice bran and fish protein hydrolysates inhibit pancreatic lipase and the mechanism of inhibition is non-competitive [45,46]. PPNs are well-known for their lipase-inhibitory activity in the gastrointestinal tract, further supporting their potential for managing dietary fat breakdown [44]. This synergy between SSPHs and PPNs could amplify their therapeutic potential. Future research could focus on elucidating the precise binding interactions between the conjugates and the enzyme, as well as their efficacy in *in vivo* models.

Considering inhibition efficiency of positive controls at 6% (Figure 3A,B), acarbose against α-amylase and α-glucosidase inhibition (92.59% and 86.43%, respectively) and orlistat inhibition against lipase activity (85.89%) (Figure 3C), A–C achieved quite similar inhibition efficiency against α-amylase, α-glucosidase and pancreatic lipase (89.29%, 81.23% and 80.69%, respectively). In conclusion, the SSPH–PPN conjugate A–C shows great promise as a natural inhibitor of α-amylase, α-glucosidase, and pancreatic lipase, achieving inhibition levels comparable to the pharmaceutical inhibitors acarbose and orlistat. This suggests that these conjugates could be further explored as functional ingredients in the development of antidiabetic and anti-obesity nutraceuticals. Furthermore, advanced analysis such as mass spectroscopy, kinetic study and molecular dynamics should be performed to elucidate the various interactions involved between inhibitors and enzymes.

Based on the enzyme inhibitory and antioxidant activities, UAH-catechin (A–C) conjugate had prominent results as compared to its counterparts, which was selected for further applications in the surimi gel formation.

### 3.6. Effect of A–C Conjugate on the Gelling Properties of Surimi Gel Obtained from Different Fish

#### 3.6.1. Breaking Force and Deformation

The breaking force (BF) and deformation force (DF) of gel samples added without and with A–C conjugate sample are presented in Table 3. Both parameters were significantly increased in a dose-dependent manner. The highest increase in BF was noticed for 6TH (290.41 ± 8.62 g) followed by 6IM gel sample (275.30 ± 3.14 g) as compared to their respective control gel samples (CTH and CIM). A similar result was noticed for DF. The differences in the BF and DF between IM and TH surimi gels can be attributed to the distinct protein compositions and functional properties of surimi derived from different fish species [46]. IM known to possess dark meat, which is associated with a higher amount of lipids and muscle-bound protease. In addition, IM also has been reported to have lower amount of the transglutaminase enzyme. Singh et al. [47] also reported the inferior gelling properties of partially purified myofibrillar proteins extracted from IM in comparison to the TB proteins. Furthermore, IM and TH have different structural proteins and amino acid profiles, influencing the gelation behavior and final gel strength [46]. The substantial rise in BF and DF was more likely due to enhanced cross-linking and improved gelation properties surimi [48]. In general, during gelation, several bondings such as covalent or non-covalent bonds could be formed during the setting of surimi paste at 40 °C. The ε-(γ-glutamyl) lysine linkages, non-disulfide covalent bond formed between myosin heavy chains by an endogenous transglutaminase, are the major bonding involved in strengthening the gel network [49]. With the addition of A–C conjugate, catechin might increase the intermolecular interactions via cross-linking, hydrogen bonding, as well as hydrophobic interactions, which contributing to stronger protein networks and higher gel strength. Zhou et al. [50] also noticed enhanced gel properties of surimi gels added with egg white modified by tea phenols at various concentrations (0.2, 0.4, 0.6, and 0.8%). Furthermore, different amino acids groups from the UAH might also favor the interactions among the myofibrillar proteins [46]. Thus, A–C conjugate synergistically improved the three-dimensional gel network of both surimi gels.

#### 3.6.2. Whiteness

The whiteness of surimi gels prepared from IM and TH, with varying levels of A–C conjugate is shown in Table 3. Irrespective of surimi types, the whiteness of gel samples was decreased with increasing concentration of the A–C. This could be associated with the dark brown color of the conjugate powder mainly affected by the catechin or UAH. Furthermore, during the setting or heating of surimi gel might lead to enzymatic browning due to the presence of protein hydrolysates and added sugars in the surimi of IM and TH surimi [51]. Additionally, the whiteness of surimi was inherently influenced by the source of extraction, which could further explain variations in color across different surimi types [18]. Similarly, Quan et al. [52] noticed a change in color upon the addition of the duck albumen hydrolysate–polyphenol conjugate in fish tofu. Among the TH and IM gels, the latter one showed lower whiteness as compared to the former, which was associated with the dark-colored flesh of the IM than the TH [53]. Furthermore, the higher amount of muscle-bound protease in the IM also resulted in the formation of smaller chain of myofibrillar proteins, which resulted in the formation of gel with irregular and weak gel network [47]. Such networks are unable to hold water, leading to lower light scattering, which causes lower whiteness [54]. The results were also supported by the lower BF of IM than TH, regardless of conjugate addition. Thus, the addition of the A–C conjugate has an impact on the whiteness of surimi gel.

#### 3.6.3. Expressible Moisture Content (EMC)

Table 3 presents the EMC of surimi gels added with varying concentrations of A–C conjugate. When compared between IM and TH surimi gels, TH showed lowest EMC, regardless of the addition of A–C at all the levels (*p* < 0.05). A lower EMC in the gel indicates that more water was effectively retained within the gel structure, leading to a stronger network with an enhanced water-holding capacity [55]. A clear decreasing trend in EMC was observed with increasing A–C levels for both types of surimi. In IM surimi, the EMC dropped from 13.8% in the control gel sample to 6.9%, when added with 6% A–C (*p* < 0.05). Similarly, in TH surimi, the EMC decreased from 11.6% in the control to 6.4% at the same level. A–C more likely interacts with the protein matrix in the surimi gels, enhancing the interconnection in gel network, which could imbibe more water. The increasing DF was also linked to the formation of higher interconnection between the myofibrillar proteins. Similar results were noticed when oxidized phenolic compounds at various concentrations (0.1 to 0.6%) were added into the *Rastrelliger kanagurta* surimi gel [11]. This finding highlights the potential of using protein–polyphenol conjugates to improve the juiciness of surimi products.

#### 3.6.4. Texture Profile Analysis

The texture profile analysis (TPA) of the surimi samples with varying concentration of A–C was conducted to assess the impact of conjugate on hardness, cohesiveness, springiness, gumminess, and chewiness of surimi samples (Table 4). It was observed that the hardness, which determines the required force to compress samples, increased with the increase in the A–C conjugate concentration in surimi gels. The lowest hardness was noticed in the control of both IM and TH surimi gels. This could be related to the formation of a gel network, which was also supported by the increasing BF (Table 3). Moreover, chewiness and gumminess followed a similar trend. In general, chewiness is the energy needed to chew the gel to the point it can be swallowed whereas gumminess is the energy needed to swallow the semisolid food [20]. Additionally, the springiness, which determines the rubberiness of the surimi gels and its ability to spring back after deformation during the first bite and cohesiveness, which the force required to overcome the internal bonding of the material, increased with increasing concentration of the A–C conjugate in both IM and TH surimi gel [18]. Therefore, the addition of the A–C conjugate in surimi gel (IM and TH) significantly improved its textural properties. The improvement in texture properties due to the incorporation of A–C in surimi allows for better structural reinforcement in surimi products. This is particularly beneficial in food applications where texture is a critical attribute for consumer preference. The enhanced hardness, cohesiveness, and chewiness suggest the potential to improve the sensory attributes of processed surimi products, making them more appealing for commercial use.

#### 3.6.5. Protein Pattern

Protein patterns of surimi gels from IM and TH are added without and with the A–C conjugate at different levels. The prominent bands of myosin heavy chain (MHC) and action of MW 206 and 48 kDa, respectively, were observed in surimi paste of both IM and TH (Figure S1). However, after gelation, MHC bands disappeared in all gel samples, indicating extensive cross-linking via endogenous transglutaminase activity [56]. This was attributed to MHC’s high susceptibility to cross-linking during the gelation process. Furthermore, proteolytic activity could also result in the disappearance of MHC band [57]. However, it confirms that the conjugate did not obstruct the formation of non-disulfide covalent bonds between myofibrillar proteins [58]. Despite the conjugate’s presence, the actin bands remained consistent across all samples, showing that actin did not act as a substrate for transglutaminase activity and, therefore, remained largely unaffected during gelation [56].

#### 3.6.6. Microstructure

**Figure 4:**
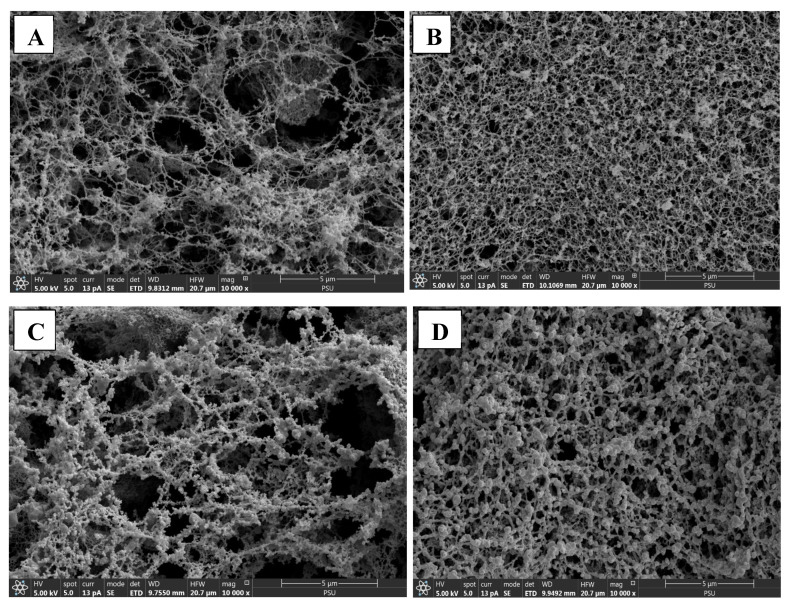
Scanning electron microscopic (SEM) images of surimi gels incorporated without and with 6% of A–C conjugate powder. (**A**) Control threadfin bream surimi gel without A–C conjugate powder; (**B**) threadfin bream surimi gel incorporated with 6% of A–C conjugate powder; (**C**) control Indian mackerel surimi gel without A–C conjugate powder; (**D**) Indian mackerel surimi gel incorporated with 6% of A–C conjugate powder.

The microstructure of selected surimi gels samples analyzed using SEM and is shown in Figure 4A–D. The control IM and TH surimi gels exhibited a looser network with large voids, reflecting their relatively lower gel strength and poor structural integrity. These larger cavities correspond to the lower breaking force observed in the control samples (Figure 4A,C), as the gel matrix was not as compact or cohesive without the A–C conjugate. In contrast, both IM and TH surimi gels with 6% A–C conjugate displayed a much denser and more interconnected protein network. The voids were significantly reduced, and the structure appeared more compact, which suggests stronger interactions between the A–C conjugate and the muscle proteins. This tighter network contributes to improved gel properties, including enhanced water retention (Figure 4B,D) and superior textural characteristics. The interpenetrating network formed by A–C conjugate and surimi allowed for better gel strength, as indicated by the increased BF compared to the control (Table 3). Thus, the addition of 6% A–C conjugate to both IM and TH surimi gels led to a more stable and compact gel matrix, improving overall gel quality.

#### 3.6.7. *In Vitro* Digestion of Surimi Gels 

After the *in vitro* digestions of surimi gels, digests were subjected to determine antioxidant and digestive enzyme inhibitory activities. 

##### Antioxidant Activity

**Figure 5:**
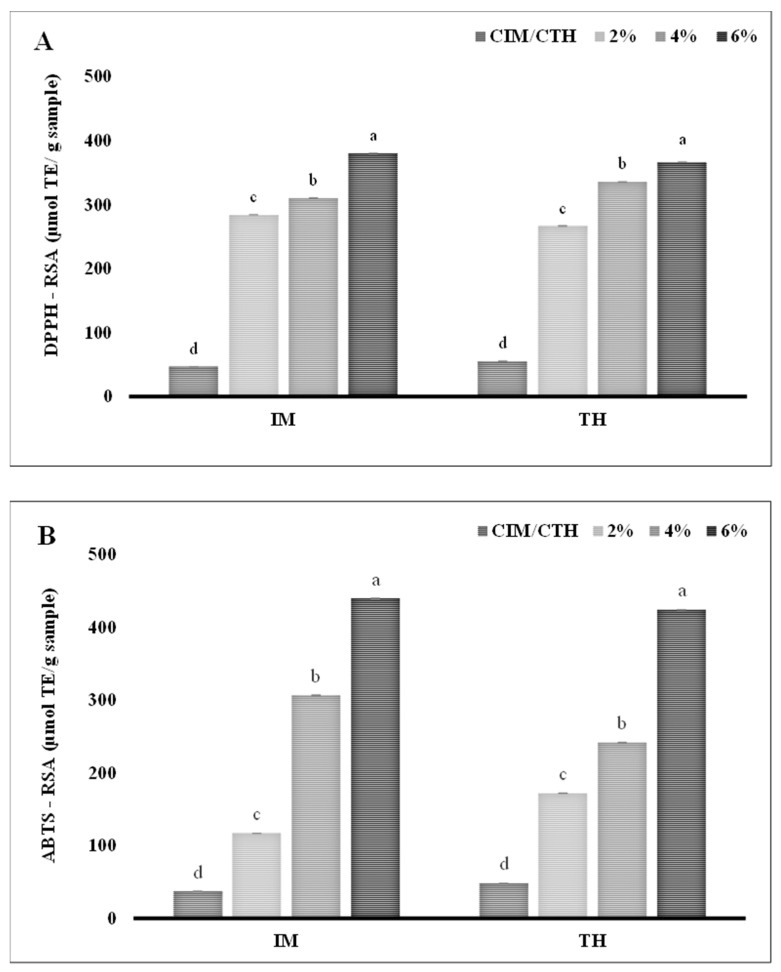
DPPH-RSA and ABTS-RSA of *in vitro* digests prepared from surimi gels incorporated with varying concentrations of UAH-catechin (**A**,**B**) conjugate. Lowercase letters on the bars indicate a significant difference in the different concentrations of the same samples (*p* < 0.05). Abbreviations: see Figure 1 and Figure 4.

The DPPH-RSA and ABTS-RSA of *in vitro* digests of IM and TH surimi gel, enriched with A–C at varying concentrations (2, 4, and 6%; *w*/*w*), are shown in Figure 5A and Figure 5B, respectively. A clear dose-dependent increase has been observed in both assays. At the highest A–C concentration (6%), the DPPH-RSA reached 379.94 μmol TE/g sample for IM surimi gel and 366.13 μmol TE/g sample for TH surimi gel. Similarly, the ABTS-RSA peaked at 6% A–C with a value of 439.44 μmol TE/g sample for IM and 423.63 μmol TE/g sample for TH, underscoring the substantial increase in free radical neutralization potential. In general, The DPPH-RSA and ABTS-RSA assays primarily evaluate the hydrogen-donating ability of antioxidants in aqueous and lipophilic environments, respectively [59]. Here, peptides derived from the SSPHs function as H-donor, stabilizing the free radicals and effectively inhibiting radical chain propagation [59]. Moreover, the presence of hydroxyl groups in catechin are known to contribute to their potent antioxidant properties, effectively neutralizing free radicals [60]. The antioxidant activity of the conjugated protein hydrolysates, underscoring synergetic effect was responsible for leading a significant increase in DPPH-RSA and ABTS-RSA of the surimi gel prepared by incorporating A–C conjugate (*p* < 0.05). These results highlight the substantial enhancement of antioxidant capacity in both surimi types with increasing A–C concentrations (*p* < 0.05). The higher DPPH-RSA and ABTS-RSA values observed in IM surimi compared to TH surimi could be attributed to differences in the protein matrices of the two fish species, which potentially affect interaction with A–C conjugate [61]. Furthermore, higher hydrolysis via muscle bound proteases in the IM more likely to produce more peptides or hydrolysates from the myofibrillar proteins. Both surimi gel types, however, demonstrated consistent, concentration-dependent antioxidant enhancement, confirming the efficiency of A–C conjugate in advancing the functional properties of surimi gels.

##### Inhibition of Digestive Enzymes

**Figure 6:**
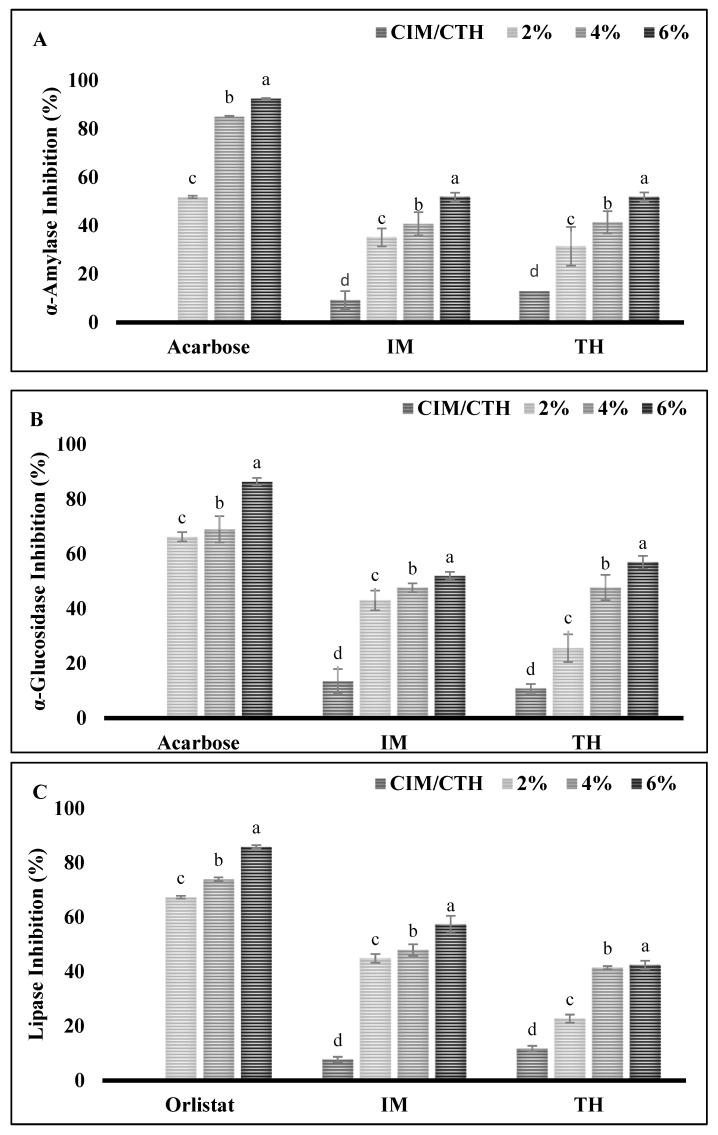
Inhibitory activity of *in vitro* digests prepared from surimi gels incorporated with varying concentrations of UAH and catechin (**A**–**C**) conjugate at various levels of various digestive enzymes such as α-amylase (**A**), α-glucosidase (**B**), and pancreatic lipase (**C**). Bars represent standard deviation (n = 3). Lowercase letters on the bars indicate a significant difference in the different concentrations of the same sample (*p* < 0.05). Abbreviations: CIM: Control Indian mackerel surimi gel, CTH: control threadfin bream surimi gel. Numbers indicate the amount added into surimi gels in percentage.

The inhibition efficiency of *in vitro* digests of IM and TH surimi incorporated with varying concentrations of A–C (2, 4, and 6%; *w*/*w*) against the α-amylase, α-glucosidase, and pancreatic lipase demonstrated a notable dose-dependent trend. As illustrated in Figure 6A–C, surimi samples with the highest A–C concentration (6%) exhibited the greatest inhibition against α-amylase, α-glucosidase, and pancreatic lipase for both IM (51.86, 51.93, and 57.31%, respectively) and TH surimi (51.85, 56.97, and 42.47%, respectively). The generation of bioactive peptides during simulated gastrointestinal digestion may contribute to a variety of beneficial activities in the surimi digest, including antioxidant and antidiabetic effects. Bioactive peptides are known for their ability to interact with digestive enzymes, potentially enhancing health benefits such as blood sugar regulation and cellular protection from oxidative stress [62]. Catechin, a polyphenolic compound found in A–C conjugates, plays a notable role in antidiabetic inhibition [38]. This pronounced enzyme inhibition suggests that the A–C conjugate interacts effectively with the enzyme active sites, potentially forming stable bonds that disrupt the catalytic process and reduce the breakdown of carbohydrates and fats. The ability of A–C-enriched surimi to inhibit α-amylase and α-glucosidase activity is particularly relevant for diabetes management, as these enzymes are directly involved in the breakdown of starches and disaccharides into glucose.

Thus, SSPH–PPNs conjugate not only enhance the gel properties of different types of surimi but also able to enhance the various activities of the conjugates.

## 4. Conclusions

The optimal concentrations for achieving the highest conjugation efficiency were, 0.5% PPN, 0.15g AcA, and 5M H_2_O_2_ at 2% (*v*/*v*), and 2% SSPH, independent to the type of polyphenol used. The conjugation of SSPHs with PPNs significantly enhances their antioxidant activities across multiple assays. Additionally, all concentrations of conjugate samples showed inhibition activity against α-amylase, α-glucosidase, and lipase; with the increase in concentration, there was a significant increase in the inhibition potential of the conjugates. Overall, the ^1^H-NMR, along with the FTIR spectrum provides compelling evidence for the successful conjugation of protein hydrolysates with EGCG, catechin, and gallic acid. Catechin and UAH conjugate showed improved gelling properties of both dark and white-fleshed fish surimi. The conjugates were able to enhance the inhibitory activity against various digestive enzymes and radicals. Thus, protein hydrolysate from shrimp industry waste could be utilized as an antidiabetic and gelling agent when combined with polyphenols. However, inhibitory mechanisms of conjugates could be elucidated via advance analysis such as molecular modeling or docking.

## Figures and Tables

**Table 1:** Effect of varying concentrations of redox pair, shrimp shell protein hydrolysates (SSPHs), and polyphenols (PPNs) on conjugation efficiency (CE) of SSP–PPN conjugates.

	UAH			UPH		
	EGCG	Catechin	Gallic Acid	EGCG	Catechin	Gallic Acid
AsA/H_2_O_2_ (%)						
0.5	75.94 ± 0.13 ^dC^	71.99 ± 0.86 ^eC^	91.52 ± 0.50 ^aC^	80.78 ± 0.42 ^cC^	75.16 ± 1.48 ^dC^	88.28 ± 1.01 ^bC^
1	79.58 ± 0.26 ^dB^	78.74 ± 1.01 ^eB^	94.17 ± 0.38 ^aB^	84.91 ± 0.47 ^cB^	77.94 ± 1.70 ^fB^	90.98 ± 0.37 ^bB^
2	83.50 ± 1.83 ^dA^	82.01 ± 0.83 ^eA^	95.41 ± 0.32 ^aA^	88.22 ± 0.12 ^cA^	83.32 ± 1.33 ^dA^	94.40 ± 1.14 ^bA^
PPNs (%)						
0.5	91.73 ± 0.40 ^cA^	90.24 ± 0.59 ^dA^	88.09 ± 0.68 ^eA^	90.22 ± 0.07 ^dA^	97.17 ± 0.16 ^aA^	96.13 ± 0.16 ^bA^
1	84.31 ± 0.56 ^eB^	89.40 ± 0.90 ^cB^	83.82 ± 0.59 ^fB^	86.64 ± 0.23 ^dB^	96.10 ± 0.26 ^aB^	95.33 ± 0.21 ^bB^
2	83.50 ± 1.83 ^dC^	88.22 ± 0.12 ^cC^	82.01 ± 0.83 ^fC^	83.32 ± 1.33 ^eC^	95.41 ± 0.32 ^aC^	94.40 ± 1.14 ^bC^
3	71.84 ± 0.20 ^fD^	74.80 ± 1.83 ^eD^	78.77 ± 1.38 ^dD^	81.05 ± 0.32 ^cD^	93.70 ± 0.40 ^aD^	91.51 ± 0.73 ^bD^
SSPHs (%)						
1	70.65 ± 0.13 ^fB^	80.58 ± 1.29 ^cB^	81.37 ± 0.06 ^bB^	72.04 ± 0.20 ^eB^	84.15 ± 0.40 ^aB^	78.19 ± 1.46 ^dB^
2	91.73 ± 0.40 ^cA^	90.24 ± 0.59 ^dA^	88.09 ± 0.68 ^eA^	90.22 ± 0.07 ^dA^	97.17 ± 0.16 ^aA^	96.13 ± 0.16 ^bA^
3	91.80 ± 0.36 ^bA^	90.96 ± 0.31 ^cA^	88.84 ± 0.15 ^dA^	90.15 ± 0.73 ^cA^	96.92 ± 0.09 ^aA^	96.58 ± 0.07 ^aA^

Values represent mean ± SD (n = 3). Different lowercase superscripts in the same row indicate significant differences (*p* < 0.05). Different uppercase superscripts in the same column indicate significant differences within the same treatment (*p* < 0.05). Abbreviations: UAH—shrimp shell protein hydrolysate prepared using alcalase; UPH—shrimp shell protein hydrolysate prepared using papain; SSPHs: shrimp shell protein hydrolysates (UAH/UPH); PPNs: plant polyphenols.

**Table 2:** Antioxidant activities of shrimp shell protein hydrolysates (SSPHs) and their different polyphenol conjugates (SSPH–PPNs).

Antioxidant Activity	UAH	UPH	A–E	A–C	A–G	P–E	P–C	P–G
* DPPH-RSA	108.67 ± 7.2 ^hY^	287.55 ± 3.43 ^gX^	661.30 ± 0.04 ^aA^	323.10 ± 0.04 ^eB^	227.09 ± 0.28 ^fB^	621.35 ± 0.17 ^bB^	584.32 ± 0.15 ^cA^	327.00 ± 0.12 ^dA^
* ABTS-RSA	556.22 ± 3.15 ^gX^	263.26 ± 0.61 ^hY^	3667.00 ± 0.06 ^aA^	2304.50 ± 0.24 ^dB^	1923.07 ± 0.28 ^eA^	2586.00 ± 0.05 ^bB^	2330.40 ± 0.13 ^cA^	1761.21 ± 0.30 ^fB^
* FRAP	18.70 ± 0.80 ^hY^	122.45 ± 0.40 ^gX^	5662.70 ± 0.30 ^aA^	4016.60 ± 2.90 ^cB^	797.90 ± 2.10 ^eA^	3777.00 ± 0.95 ^dB^	4610.00 ± 2.70 ^bA^	693.00 ± 0.75 ^fB^
# MCA	12.73 ± 2.20 ^gX^	4.54 ± 0.25 ^hY^	2449.00 ± 2.10 ^aA^	2028.00 ± 2.40 ^dB^	1591.30 ± 2.40 ^eA^	2230.00 ± 2.40 ^bB^	2122.60 ± 0.23 ^cA^	1498.60 ± 1.20 ^fB^

Value represents ± SD (n = 3). Different lowercase superscripts in the same row indicate significant differences (*p* < 0.05). Different uppercase superscripts (A and B) in the same row indicate significant differences within different hydrolysates of the same polyphenol (*p* < 0.05). Different uppercase superscripts (X and Y) in the same row indicate significant differences between UAH and UPH (*p* < 0.05). Abbreviations: see Table 1. * μmol TE/g solid. # μmol EE/g solid.

**Table 3:** Breaking force, deformation, whiteness, and expressible moisture content of Indian mackerel and threadfin bream surimi gels added without and with A–C conjugate at various concentrations.

	Breaking Force (BF; g)	Deformation (DF; mm)	Whiteness	EMC (%)
Indian mackerel (IM)				
CIM	150.20 ± 5.10 ^d^	11.40 ± 0.79 ^c^	62.60 ± 0.09 ^a^	13.80 ± 0.2 ^a^
2IM	215.60 ± 7.19 ^c^	13.30 ± 0.16 ^b^	58.71 ± 0.25 ^b^	10.00 ± 0.6 ^b^
4IM	245.40 ± 0.14 ^b^	13.89 ± 0.03 ^b^	56.68 ± 0.13 ^c^	9.20 ± 0.01 ^c^
6IM	275.30 ± 3.14 ^a^	15.21 ± 0.90 ^a^	54.33 ± 0.01 ^d^	6.90 ± 0.23 ^d^
Threadfin bream (TH)				
CTH	135 ± 0.11 ^d^	10.11 ± 1.13 ^c^	69.84 ± 0.03 ^a^	11.60 ± 0.30 ^a^
2TH	220.90 ± 6.2 ^c^	12.61 ± 0.32 ^b^	65.75 ± 0.04 ^b^	9.50 ± 0.62 ^b^
4TH	255.50 ± 3.4 ^b^	14.56 ± 0.85 ^a^	63.94 ± 0.31 ^c^	8.70 ± 0.10 ^c^
6TH	290.41 ± 8.62 ^a^	14.98 ± 0.03 ^a^	60.71 ± 0.01 ^d^	6.40 ± 0.15 ^d^

Values represent mean ± SD (n = 3). Different lowercase superscripts in the same column indicate significant differences (*p* < 0.05).Abbreviations: CIM and CTH: Surimi gel from Indian mackerel and threadfin bream surimi gel added without A–C conjugate powder; 2IM, 4IM and 6IM: Indian mackerel surimi gels incorporated with 2, 4, and 6% A–C conjugate powder, respectively; 2TH, 4TH, and 6TH: threadfin bream surimi gels incorporated with 2, 4, and 6% A–C conjugate powder, respectively.

**Table 4:** Texture profile analysis of Indian mackerel and threadfin bream surimi gels added without and with A–C conjugate at various concentrations.

	Hardness (g)	Cohesiveness (g)	Springiness (cm)	Gumminess (g)	Chewiness (g.cm)
Indian mackerel					
CIM	483.47 ± 6.21 ^d^	0.42 ± 0.06 ^d^	0.16 ± 0.02 ^d^	86.67 ± 5.84 ^d^	34.00 ± 1.47 ^d^
2IM	816.90 ± 17.31 ^c^	0.85 ± 0.05 ^c^	0.25 ± 0.09 ^c^	460.00 ± 10.00 ^c^	370.00 ± 10.00 ^c^
4IM	861.97 ± 7.68 ^b^	0.96 ± 0.01 ^b^	0.28 ± 0.01 ^b^	490.00 ± 10.00 ^b^	400.00 ± 10.00 ^b^
6IM	905.45 ± 10.40 ^a^	0.95 ± 0.01 ^a^	0.29 ± 0.04 ^a^	470.00 ± 0.40 ^a^	475.19 ± 6.90 ^a^
Threadfin bream					
CTH	893.87 ± 7.79 ^d^	0.78 ± 0.08 ^d^	0.23 ± 0.01 ^d^	307.08 ± 15.12 ^d^	230.47 ± 30.27 ^d^
2TH	962.38 ± 7.78 ^c^	0.91 ± 0.01 ^c^	0.26 ± 0.05 ^c^	340.00 ± 12.01 ^c^	270.00 ± 10.00 ^c^
4TH	984.67 ± 4.09 ^c^	0.94 ± 0.01 ^b^	0.31 ± 0.01 ^b^	380.00 ± 10.33 ^b^	300.00 ± 10.00 ^b^
6TH	998.84 ± 7.09 ^a^	0.98 ± 0.08 ^a^	0.33 ± 0.05 ^a^	387.08 ± 9.16 ^a^	350.17 ± 9.27 ^a^

Values represent mean ± SD (n = 3). Different lowercase superscripts indicate significant differences in the same columns (*p* < 0.05). Abbreviations: see Table 3.

## Data Availability

The original contributions presented in this study are included in the article/Supplementary Materials. Further inquiries can be directed to the corresponding author.

## Supplementary Materials

The following supporting information can be downloaded at: https://www.mdpi.com/article/10.3390/foods13244022/s1, Figure S1: Protein pattern of surimi gels incorporated without and with varying concentration of A-C (2, 4, and 6%).
