# Supplementary material for: Enhanced Antioxidant and Digestive Enzyme Inhibitory Activities of Pacific White Shrimp Shell Protein Hydrolysates via Conjugation with Polyphenol: Characterization and Application in Surimi Gel
Source: Foods. 2024 Dec 12;13(24):4022. doi: 10.3390/foods13244022 (PMC11728318; doi:10.3390/foods13244022)
Supplement: Supplementary file 1 [file foods-13-04022-s001.zip › foods-3341852-supplementary.pdf]

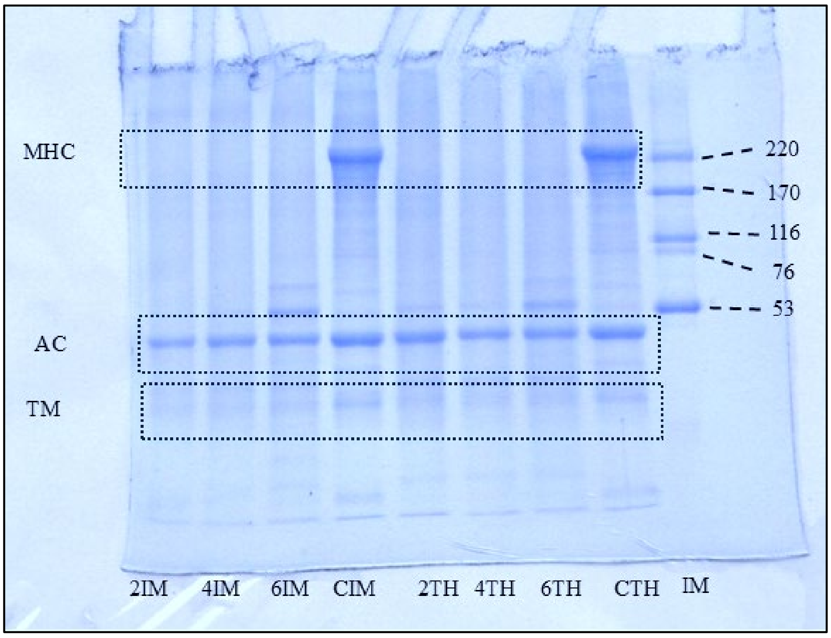

Figure S1. Protein pattern of surimi gels incorporated without and with varying concentration of A-C (2, 4, and 6%). Caption: CIM: control of Indian mackerel surimi paste without A-C conjugate powder, 2IM 2% A-C, IM Indian mackerel, 4IM 4% A-C, IM Indian mackerel, 6IM : 6% A-C, IM Indian mackerel), CTH: control of threadfin surimi paste without A-C conjugate powder, TH: Threadfin bream, 2 TH : 2% A-C,TH Threadfin Bream, 4 TH : 4% A-C,TH Threadfin Bream, 6 TH : 6% A-C, TH Threadfin Bream.
